# Modulation of Triglyceride and Cholesterol Ester Synthesis Impairs Assembly of Infectious Hepatitis C Virus*[Fn FN2]

**DOI:** 10.1074/jbc.M114.582999

**Published:** 2014-06-10

**Authors:** Jolanda M. P. Liefhebber, Charlotte V. Hague, Qifeng Zhang, Michael J. O. Wakelam, John McLauchlan

**Affiliations:** From the ‡ Medical Research Council-University of Glasgow Centre for Virus Research, 8 Church Street, Glasgow G11 5JR, Scotland, United Kingdom and; § The Babraham Institute, Babraham Research Campus, Cambridge CB22 3AT, United Kingdom

**Keywords:** Cholesterol, Hepatitis C Virus (HCV), Hepatitis Virus, Lipid Droplet, Triglyceride, Virus Assembly

## Abstract

In hepatitis C virus infection, replication of the viral genome and virion assembly are linked to cellular metabolic processes. In particular, lipid droplets, which store principally triacylglycerides (TAGs) and cholesterol esters (CEs), have been implicated in production of infectious virus. Here, we examine the effect on productive infection of triacsin C and YIC-C8-434, which inhibit synthesis of TAGs and CEs by targeting long-chain acyl-CoA synthetase and acyl-CoA:cholesterol acyltransferase, respectively. Our results present high resolution data on the acylglycerol and cholesterol ester species that were affected by the compounds. Moreover, triacsin C, which blocks both triglyceride and cholesterol ester synthesis, cleared most of the lipid droplets in cells. By contrast, YIC-C8-434, which only abrogates production of cholesterol esters, induced an increase in size of droplets. Although both compounds slightly reduced viral RNA synthesis, they significantly impaired assembly of infectious virions in infected cells. In the case of triacsin C, reduced stability of the viral core protein, which forms the virion nucleocapsid and is targeted to the surface of lipid droplets, correlated with lower virion assembly. In addition, the virus particles that were released from cells had reduced specific infectivity. YIC-C8-434 did not alter the association of core with lipid droplets but appeared to decrease production of infectious virus particles, suggesting a block in virion assembly. Thus, the compounds have antiviral properties, indicating that targeting synthesis of lipids stored in lipid droplets might be an option for therapeutic intervention in treating chronic hepatitis C virus infection.

## Teaser

**Background:** Lipid droplets have been implicated in HCV virion assembly.

**Results:** Inhibitors of the synthesis of triacylglycerol and cholesterol ester, the main components of lipid droplets, impair virion assembly.

**Conclusion:** Production of infectious HCV is linked integrally with the biosynthesis of the major components of lipid droplets.

**Significance:** Inhibitors of enzymes that generate acylglycerols and cholesterol esters have antiviral activity.

## Introduction

Hepatitis C virus (HCV)^3^ typically establishes a chronic infection in the liver that can lead to a range of hepatic disorders including cirrhosis, hepatocellular carcinoma, and liver failure (1). Metabolic diseases including insulin resistance and steatosis are particular features of chronic infection (2, 3), and there is now conclusive evidence that factors and pathways involved in lipid metabolism are interlinked with various stages of the virus life cycle (4). First, virus particles are found in association with lipoprotein in serum, forming lipoviroparticles that are infectious in animal models (5–7). Second, the low density lipoprotein receptor and SRB-1, a scavenger receptor for cholesterol, contribute to virus entry (8, 9). Third, HCV RNA replication is sensitive to lipid-modulating agents, and finally, virion assembly is thought to be coupled to the assembly pathway for very low density lipoprotein (VLDL) (10–14). Within this pathway, storage organelles called lipid droplets (LDs) play a crucial role in the production of lipoprotein particles (15–17).

Much of the evidence for a role for LDs in HCV virion assembly has focused on the viral core protein, which is targeted to the surface of the organelles after maturation by two host proteases, signal peptidase and signal peptide peptidase (18–22). The mature form of core can be separated into two domains: the D1 domain, which has a high proportion of positively charged residues and binds RNA, and the D2 domain, which consists of two amphipathic α-helices separated by a hydrophobic loop (23, 24). Mutations in the D2 domain that disrupt LD attachment reduce the yield of infectious virus (25–27). Other viral proteins (*e.g.* NS5A and NS3) are also found in close proximity to or bound to the surface of LDs in cells producing virions (26, 28–30). Moreover, some LDs with attached viral proteins are juxtaposed to the sites of HCV RNA replication (25, 26). It has been proposed that such close apposition of replication sites and LDs coated with viral proteins may indicate sites where the first stages of virion assembly occur. Because LDs participate in the lipidation of VLDL and infectious virions are associated with lipoprotein, the targeting of LDs by viral proteins could represent a mechanism for the virus to access the VLDL assembly pathway.

Although there is evidence for the participation of LDs in HCV assembly, how they contribute to virion production has not been fully elucidated. Previously, we have shown that disrupting the redistribution of LDs, which is mediated by the HCV core protein and requires trafficking by the microtubule network, reduces virus production (25). Other approaches to establish the role of LDs in virion assembly have relied on targeting cellular factors that are involved in lipid metabolism. For example, a recent report has shown that nordihydroguaiaretic acid, a hypolipidemic drug that represses fatty acid production while stimulating fatty acid oxidation (31), suppresses virus release (32). The mechanism involved in this suppression was suggested as resulting from an increase in the average size of LDs that was accompanied by an overall decrease in their number. The drug also inhibited VLDL secretion apparently by inducing a decrease in transcription of the microsomal triglyceride transfer protein gene. Compared with the broad spectrum of genes controlling fatty acid metabolism that are affected by nordihydroguaiaretic acid, specific targeting of diacylglycerol acyltransferase 1 (DGAT1), which is responsible for the final step in triglyceride synthesis, impairs virion production (33). From this study, it has been proposed that virus assembly requires LDs generated through DGAT1 activity.

The lipid composition of LDs is distinct between the surface and the core of the organelles. The LD core is primarily made up of triglycerides (TAGs) and cholesterol esters (CEs), although diacylglycerides (DAGs) are also incorporated into LDs; the presence of other lipid species such as free cholesterol and fatty acids in the LD core cannot be excluded (34–37). Phospholipids predominate at the LD surface, mainly phosphatidylcholine, but phosphatidylethanolamine, phosphatidylinositol, cholesterol, lysophosphatidylcholine, and lysophosphatidylethanolamine are also detected (34). Interestingly, the phospholipid monolayer at the surface of LDs has a distinct fatty acid composition that differs from that for the endoplasmic reticulum (38).

Here, we focused on deepening insight into the role of LDs in HCV infection by modulating the intracellular synthesis of the two major components of droplets, namely TAGs and CEs, using two compounds, triacsin C and YIC-C8-434. Triacsin C is a potent inhibitor of long-chain acyl-CoA synthetase, an enzyme that generates fatty acyl-CoAs for incorporation into triglycerides and cholesterol esters (39). Conversely, YIC-C8-434 blocks the conversion of cholesterol to cholesterol esters through inhibition of Acyl-CoA:cholesterol acyltransferase (40). Our study examined the impact of these compounds on the cellular lipidome and distribution of LDs and hence their effects on HCV RNA replication and virion assembly.

## EXPERIMENTAL PROCEDURES

### 

#### 

##### Reagents

YIC-C8-434 and triacsin C were obtained from Sigma-Aldrich and Enzo Life Sciences, respectively. Chemicals for lipid extraction (HPLC grade) were purchased from Fisher Scientific, and lipid standards were supplied by Avanti Polar Lipids with the exception of TAGs, MAGs, and free fatty acids, which were obtained from Sigma-Aldrich. The pJFH1 plasmid and LD540 were gifts from Takaji Wakita (National Institute of Infectious Diseases, Tokyo, Japan) and Christoph Thiele (University of Bonn), respectively. Antibodies used to detect HCV core (rabbit antiserum 4210), E2 (AP33; a gift from Arvind Patel, Glasgow University), dsRNA (J2; supplied by SCICONS, Hungary), NS5A (sheep antiserum; a gift from Mark Harris, Leeds University), NS3 (mouse antibody; a gift from Thomas Pietschmann, TWINCORE, Hannover, Germany), human apoE (clone EP1374Y; supplied by Abcam), and human Perilipin-2 (PLIN2) have been described previously (25, 41–44). For detection of PLIN2 by indirect immunofluorescence, an antibody raised in guinea pig against the protein was used (Progen). Actin was detected in Western blots with an antibody purchased from Sigma.

##### Maintenance of Tissue Culture Cells

Huh-7 cells were propagated in Dulbecco's modified Eagle's medium (DMEM) supplemented with 10% fetal calf serum and 100 IU/ml penicillin-streptomycin.

##### In Vitro Synthesis of RNA, Electroporation of Cells, and Treatment with Compounds

Plasmid pJFH1, which contains the full-length genomic sequence for HCV strain JFH1 (genotype 2a), was linearized with XbaI and treated with mung bean nuclease. RNA was transcribed *in vitro* from linearized DNA using the T7 RiboMAX Express Large Scale RNA Production System (Promega). Transcripts were introduced into Huh-7 cells by electroporation as described previously (45). Two days after electroporation with JFH1 RNA, cells were incubated with the appropriate concentrations of triacsin C and YIC-C8-434 for 5 h followed by a further 24 h with fresh medium containing the compounds. At the end of treatment, culture medium and cells were harvested for further analysis.

##### Concentration of Virus Particles

Virus particles in ∼30 ml of culture medium from infected cells were concentrated by filtration through a 0.45-μm filter followed by centrifugation through a 3-ml cushion of 20% sucrose. Virus particles were pelleted at 100,000 × *g* for 5 h at 4 °C. Pellets were dissolved in sample buffer used for SDS-PAGE (see below).

##### Infection of Cells and Determination of Virus Titers

Supernatants from cells electroporated with JFH1 RNA were removed at required time points and used to infect monolayers of naive Huh-7 cells. Infected cells were detected at 3 days postinoculation by indirect immunofluorescence using NS5A antiserum. The tissue culture 50% infectious dose (TCID_50_) was determined by limiting dilution assay (46). For measuring the TCID_50_ of intracellular virus, cells were washed with phosphate-buffered saline (PBS), scraped into PBS, and pelleted by centrifugation at 400 × *g* for 5 min at room temperature. Pelleted cells were resuspended in fresh culture medium, and after several cycles of freeze-thawing, cell debris was removed by centrifugation at 1500 × *g* for 5 min. The supernatant of the cell suspension was used to infect Huh-7 cells to determine the TCID_50_.

##### Preparation of Cell Extracts, SDS-PAGE, and Western Blot Analysis

To prepare extracts, cell monolayers were washed with PBS and solubilized in sample buffer (160 mm Tris-HCl, pH 6.7, 2% SDS, 700 mm β-mercaptoethanol, 10% glycerol, bromophenol blue). Samples were heated at 95 °C for 10 min prior to electrophoresis through 8, 10, or 12% SDS-polyacrylamide gels (acrylamide:bisacrylamide ratio of 37.5:1). For Western blot analysis, proteins were transferred to nitrocellulose membrane. After blocking with blocking buffer (PBS containing 5% milk powder (Marvel) and 0.1% Tween 20), membranes were incubated with the relevant primary and HRP-linked secondary antibodies diluted in blocking buffer. After washing, bound antibody was detected using ECL Plus (Amersham Biosciences).

##### Indirect Immunofluorescence Staining and Quantification of Lipid Droplets

Electroporated cells plated on 13-mm coverslips were fixed in methanol at −20 °C for 20 min or in 4% paraformaldehyde for 1 h at room temperature. After paraformaldehyde fixation, cells were permeabilized with 40 μg/ml digitonin for 30 min at room temperature. Cells were washed in PBS and then incubated with primary antibody diluted in PBS/FCS (PBS containing 1% fetal calf serum) for 2 h at room temperature. Cells were washed extensively with PBS and then incubated with secondary antibody conjugated to Alexa Fluor fluorescent tags diluted in PBS/FCS for 60 min at room temperature. After washing with PBS, cells were rinsed with distilled water before mounting on slides using water and sealed with nail polish. To stain LDs, cells were fixed with 4% paraformaldehyde in PBS for a minimum of 1 h followed by washing with PBS. The cells were then incubated with a 1:2000 dilution of LD540 (stock solution, 0.5 mg/ml in ethanol) in PBS for 30 min. After washing with PBS, cells were rinsed with distilled water before mounting on slides using water and sealed with nail polish. Slides were thereafter examined with a Zeiss LSM510 META microscope. Images were recorded with a Plan-Apochromat ×63 lens (numerical aperture, 1.4). Adobe Photoshop (CS2) was used for further image processing.

To quantify the numbers of LDs and estimate their sizes, images of cells stained with LD540 were converted to black and white pictures, and the same threshold was applied to cell images using WCIF ImageJ (open source NIH). Cell areas were manually drawn, and this region of interest was used to analyze and calculate average LD size and number in each cell with ImageJ software.

##### Buoyant Density Fractionation of HCV Virions

Virions from the culture medium were concentrated by filtration through a 0.45-μm filter followed by centrifugation at 100,000 × *g* for 5 h at 4 °C. The pellet was dissolved overnight in PBS at 4 °C and applied to the top of a continuous 10–40% iodixanol gradient. After centrifugation at 200,000 × *g* at 4 °C for 16–18 h, fractions were taken from the top and stored at 4 °C for later analysis of virus titers by measuring secreted embryonic alkaline phosphatase from Huh-7/J20 cells as described previously (47). In short, Huh-7/J20 cells were seeded the day before infection with the gradient fractions. The cells were incubated for 3 days at 37 °C after infection with each fraction (in triplicate). Cells were then lysed with a buffer containing 50 mm Tris-HCl, pH 7.4, 0.1 m NaCl, 1 mm EDTA, and 0.5% Triton X-100. Placental alkaline phosphatase secretion was measured using the Phospha-Light System from Applied Biosystems according to the manufacturer's instructions.

##### Statistical Analysis

Unpaired *t* tests were performed in all cases where statistical validation was required.

##### Real Time Quantification of RNA

Total cellular and viral RNA was extracted from Huh-7 cells using the RNeasy Mini kit (Qiagen) according to the manufacturer's instructions. RT-qPCRs were carried out in two stages. First, cDNA was produced from total RNA using a TaqMan kit (Applied Biosciences) and random hexamer oligonucleotides following the manufacturer's instructions. Reverse transcription was performed in a Thermo Hybaid PX2 thermal cycler using the following conditions: (i) primer annealing at 25 °C for 10 min, (ii) strand elongation at 37 °C for 1 h, and (iii) reverse transcriptase inactivation at 95 °C for 5 min. For the second step, cDNAs were analyzed by qPCR using TaqMan Fast Universal PCR Master Mix, no AmpErase UNG (Applied Biosystems). Each sample was run as a singleplex reaction containing appropriate primers and TaqMan probe or probe/primer mixture. The HCV probe and primer sequences were located in the 5′-UTR of the JFH1 genome (primers, 5′-TCTGCGGAACCGGTGAGTAC-3′ (nucleotides 147–166) and 5′-GCACTCGCAAGCACCCTATC-3′ (nucleotides 295–314); probe, 5′-GGCCTTGTGGTACTG-3′ (nucleotides 277–291)). GAPDH probe/primer mixture (VIC/minor groove binder probe) was purchased from Applied Biosystems. Reactions were performed using an Applied Biosystems 7500 Fast Real-Time PCR machine as follows: (i) dsDNA strand separation at 95 °C for 3 s and (ii) primer annealing and strand elongation at 60 °C for 30 s. Steps i and ii were repeated 40 times. All qPCRs were performed in triplicate, and values were normalized to endogenous GAPDH levels. Data were analyzed using 7500 Fast System Software (SDS version 1.3.1, Applied Biosystems).

##### Lipid Extraction from Cells

Subconfluent Huh-7 cells on 150-mm culture dishes were treated either with DMSO, triacsin C, or YIC-C8-434 for a total of 29 h at 37 °C. Growth medium was then removed, and the cells were washed twice with PBS. Cells were scraped into 5 ml of PBS and pelleted at 500 × *g* for 10 min. Cell pellets were typically stored for short periods at −20 °C prior to extraction of lipid. To extract lipids, cell pellets were resuspended in 0.75 ml of ice-cold methanol and transferred to silanized screw-capped glass tubes. A further 0.75 ml of methanol was used to rinse the container and combined with the initial volume. Internal standards were then added to allow quantification of lipid species. To this solution, 1.5 ml of 0.9% NaCl and 3 ml of chloroform were added to give a final proportion of 2:1:1 chloroform:methanol:water (Folch procedure). This mixture was vortexed vigorously to mix the organic and aqueous phases and then sonicated in an ice-cooled ultrasonic bath for 5 min. After a second vortex, the tubes were centrifuged at 1000 × *g* for 15 min to separate aqueous and organic phases. The lower organic phase was transferred into a silanized glass tube using a glass Pasteur pipette. The upper phase was re-extracted as before with 3 ml of chloroform. Both lower phases were then combined, and the chloroform was evaporated. Samples were frozen at −20 °C for short term storage before analysis by mass spectrometry.

##### Mass Spectrometry and Determination of Lipids

A Shimadzu ion trap-TOF LC-MS/MS system hyphenated with a five-channel on-line degasser, four pumps, column oven, and autosampler with cooler (Prominence HPLC, Shimadzu) was used for lipid analysis. The lipid classes were separated using a normal phase silica gel column (2.1 × 150 mm, 4 μm, MicroSolv Technology) with hexane/dichloromethane/chloroform/methanol/acetonitrile/water/ethylamine solvent gradients (48) based on the polarity of the headgroup. Accurate mass (mass accuracy of ∼5 ppm) and tandem mass spectrometry (MS) were used for molecular species identification and quantification. The identity of lipids was further confirmed by using appropriate lipid standards. The ion trap-TOF MS operation conditions were: ESI interface voltage, +4.5 kV for positive ESI and −4 kV for negative ESI; heat block temperature, 230 °C; nebulizing gas flow, 1.4 liters/min; curved desolvation line temperature, 210 °C with drying gas on at a pressure of 100 kilopascals. All the solvents used for lipid extraction and LC-MS/MS analysis were of LC-MS grade (Fisher Scientific). For cholesterol (CH) and CE analysis, we modified the method of Liebisch *et al.* (49). Briefly, lipid extracts remaining from the other lipid analyses were dried at room temperature using a Thermo SpeedVac. The dried lipid residues were acetylated with 170 ml of 1:5 (v/v) acetyl chloride:chloroform at room temperature for 2 h. The acetylation solvent was removed at room temperature under vacuum. The acetylated residues were redissolved in 40 μl of 10 mm ammonium acetate in 3:1 (v/v) methanol:chloroform, and 7 μl was injected via an autosampler for ESI-MS/MS analysis. A Shimadzu Prominence HPLC hyphenated with ABSciex 4000 QTRAP was used for CH and CE analysis; 7.5 mm ammonium acetate in 3:1 (v/v) methanol:chloroform (0.25 ml/min) was pumped to the 4000 QTRAP source for ESI-MS/MS analysis. The following MS operation parameters were used: (i) source/gas parameters: curtain gas, 20; collision gas, medium; ion spray voltage, 5500 V; temperature, 400 °C; ion source gas 1, 45; ion source gas 2, 20; interface heater, on; (ii) compound parameters: entrance potential, 9.0; collision cell exit potential, 11.0; declustering potential, 60 for CE analysis, 50 for acetylated CH analysis; collision energy, 19 for CE analysis, 15 for acetylated CH analysis. Both Q1 and Q3 masses were set at unit resolution for multiple reaction monitoring analysis of each molecular species of CE and CH. For the detailed multiple reaction monitoring setup, see Liebisch *et al.* (49).

## RESULTS

### 

#### 

##### Changes in Intracellular Lipid Composition Elicited by Triacsin C and YIC-C8-434

In this study, we examined the roles of LDs and lipid metabolism in HCV infection using compounds that modulate synthesis of acylglycerides and CEs, the major components of LDs. Two compounds were selected for this purpose, triacsin C and YIC-C8-434. Based on cell viability experiments across a range of concentrations for both compounds, Huh-7 cells had 85% viability after incubation for 29 h with 0.15 and 0.3 μm triacsin C and 10 and 20 μm YIC-C8-434 (supplemental Fig. 1); at higher concentrations, cell viability decreased to less than 80% (data not shown). Therefore, these subtoxic concentrations were selected for studies on their effects on intracellular lipids, LDs, and the HCV life cycle.

**FIGURE 1.**
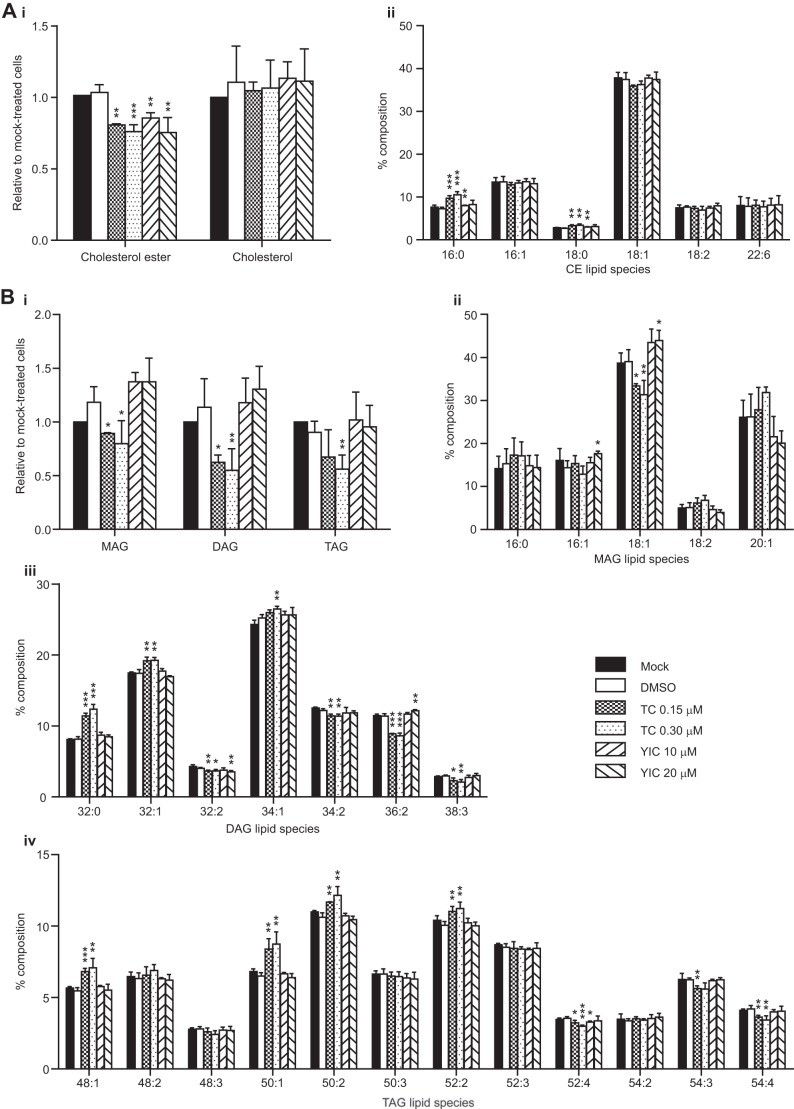
**Changes in lipid composition brought about by triacsin C and YIC-C8-434.** Lipids were extracted from control and inhibitor-treated cells, and the lipid composition was determined by HPLC-ESI-MS. *A* and *B*, *panel i*, the relative total amounts of CEs, CH, MAGs, DAGs, and TAGs in DMSO-, triacsin C- (*TC*), or YIC-C8-434 (*YIC*)-treated cells compared with control cells. *A*, *panel ii*, and *B*, *panels ii–iv*, the molecular species of each of lipid class was determined and expressed as a percentage of the total of the species within a lipid class. *Error bars* represent S.D. of three determinations. * represents *p* < 0.1, ** is *p* < 0.05, and *** is *p* < 0.01.

To determine the alterations in lipid composition following treatment with the two inhibitors, extracts from cells treated with triacsin C and YIC-C8-434 were analyzed by HPLC-MS (Fig. 1). Triacsin C reduced the amount of cellular MAGs, DAGs, TAGs, and CEs but had no effect on cholesterol levels (Fig. 1, *A* and *B*, *panel i* in each case). YIC-C8-434 reduced the level of CEs, and we observed accompanying rises in cholesterol as well as MAGs and DAGs, although these did not reach statistical significance (Fig. 1, *A* and *B*, *panel i*). Examination of the molecular species of the neutral lipids indicated a selective effect of triacsin C because not all molecular species were affected equally. Notably, the amounts of 18:1 MAG and its likely acylated product 36:2 DAG (Fig. 1*B*, *panels ii* and *iii*) were reduced. The TAG species were generally reduced, although the minor species TAG 48:1, TAG 50:1, TAG 50:2, and TAG 52:2 appeared to be unaffected as their relative proportions were significantly elevated (Fig. 1*B*, *panel iv*). Fig. 1*A* (*panel ii*) shows no overall change in most CE molecular species following triacsin C and YIC-C8-434 treatment except for small but significant increases in the 16:0 and 18:0 CE species by triacsin C (both concentrations) and YIC-C8-434 at 10 μm. These subtle alterations require more detailed analysis to determine the apparent selectivity induced by the compounds.

##### Morphologic Changes to Lipid Droplets following Treatment with Triacsin C and YIC-C8-434

**FIGURE 2.**
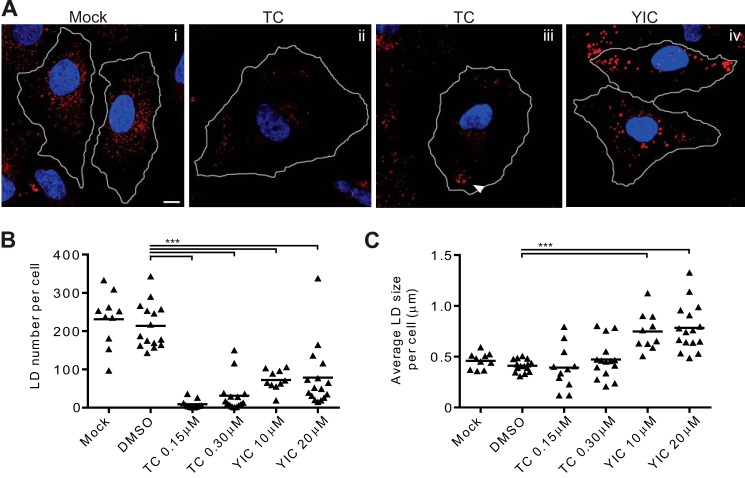
**Morphologic changes to LDs after treatment with triacsin C and YIC-C8-434.** *A*, Huh-7 cells were mock-treated (*panel i*) or incubated with 0.15 or 0.3 μm triacsin C (*TC*) (*panels ii* and *iii*, respectively) or 10 μm YIC-C8-434 (*YIC*) (*panel iv*) for 24 h followed by fixation and staining for LDs and nuclei using LD540 (*red*) and DAPI (*blue*), respectively. LDs in the periphery of the cell are indicated with an *arrowhead*. The *scale bar* in *panel i* represents 10 μm. The number of LDs (*B*) and their average size in μm (*C*) per cell was calculated in at least 10 cells for each treatment. Each *triangle* represents one cell and indicates its respective number or average size of lipid droplets. The overall average of each condition is shown as a *line*. *** represents a *p* value of <0.001 in *B* and <0.005 in *C*.

To further assess the impact of triacsin C and YIC-C8-434, immunofluorescence was used to determine their effect on the abundance, size, and distribution of LDs. Huh-7 cells were treated with both compounds for 29 h, then fixed, and stained for LDs using LD540, a lipophilic dye (50). In agreement with previous reports, mock-treated Huh-7 cells contained abundant numbers of LDs that were dispersed throughout the cell (Fig. 2*A*, *panel i*). A similar pattern was observed for cells treated with DMSO (data not shown). However, cells incubated with triacsin C were almost completely devoid of visible LDs (Fig. 2*A*, *panels ii* and *iii*); those that could be detected tended to cluster toward the cell periphery (Fig. 2*A*, *panel iii*, *arrow*, and supplemental Fig. 2). In contrast to the effect of triacsin C, the LDs in cells treated with YIC-C8-434 were larger than those in mock-treated cells (Fig. 2*A*, *panel iv*). To provide a more quantitative assessment of the effects of the compounds, we also conducted detailed analysis of overall numbers and sizes of stained LDs in drug-treated cells (Fig. 2, *B* and *C*). The data revealed a significant decrease in the number of LDs in both triacsin C- and YIC-C8-434-treated cells with triacsin C-treated cells containing very few LDs (Fig. 2*B*). For those cells that retained some LDs after triacsin C treatment, the average size of LDs did not differ significantly from mock-treated cells (Fig. 2*C*). However, LDs in YIC-C8-434-treated cells were on average twice the size of those in mock-treated cells but could be up to 3 times larger (Fig. 2*C*). Combined with the overall decrease in the number of stained LDs in cells treated with YIC-C8-434, the compound may have induced a modest increase in lipid stored in LDs. This additional stored lipid could be derived from the greater abundance of cholesterol, MAGs, and DAGs that is detected in YIC-C8-434-treated cells. In addition, there may be increased targeting of cholesterol and acylglycerols from other cellular compartments to LDs. From these data, we concluded that triacsin C and YIC-C8-434 had quite distinct effects on LDs. Blocking synthesis of acylglycerides and CEs with triacsin C significantly decreased the numbers of LDs, whereas impairing the conversion of CH to CEs with YIC-C8-434 led to an increase in the size of LDs.

##### Triacsin C and YIC-C8-434 Impair Virus Production

**FIGURE 3.**
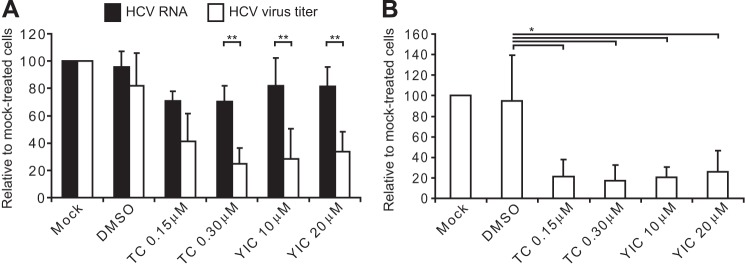
**Virus production is impaired by triacsin C and YIC-C8-434.** Huh-7 cells were electroporated with JFH1 RNA and 48 h later mock-treated or incubated with DMSO, 0.15 or 0.3 μm triacsin C (*TC*), or 10 or 20 μm YIC-C8-434 (*YIC*) for 29 h. Cells were lysed and used for RNA isolation and RT-qPCR to measure relative HCV RNA levels in each condition (*A*, *black bars*). Culture medium from the last 24 h was used to determine virus titers. These were plotted relative to mock-treated cells (*A*, *white bars*). ** indicates *p* < 0.05 between the relative values of HCV RNA and virus titers calculated from five independent experiments. In *B*, cells were subjected to several freeze-thaw cycles to analyze intracellular virus titers from three independent experiments and assessed relative to mock-treated cells. *p* < 0.1 is indicated by *. *Error bars* in *A* and *B* represent S.D.

Previous reports have shown that compounds that block the early stages of lipid biosynthesis decrease HCV replication (11, 12, 51, 52). The effect of triacsin C and YIC-C8-434 on viral RNA synthesis and virion production was tested using the HCVcc system, which generates infectious virus that is released from cells. Huh-7 cells were electroporated with full-length genomic JFH1 RNA and then treated with either compound at 48 h after electroporation for a total of 29 h. Relative quantification of viral RNA in infected cells following drug treatment revealed that triacsin C reduced HCV transcripts by just over 20% (Fig. 3*A*). For YIC-C8-434, the drop in viral RNA levels was less marked (Fig. 3*A*). In contrast to the modest reduction in viral RNA levels in cells treated with triacsin C, there was a greater decrease in virus released from cells that reached 3-fold at the higher concentration of the drug (Fig. 3*A*). At this concentration of triacsin C, the reduction in virus production was significantly different from any effect of the drug on viral RNA synthesis. YIC-C8-434 lowered the titer of infectious virus to a similar extent as triacsin C, and there was a significant difference between the effect on HCV RNA replication and virion production (Fig. 3*A*).

Measurement of extracellular virus assesses both intracellular assembly and subsequent release of virions from infected cells. To examine whether the reduction in infectious virus arises from either assembly or release, cells electroporated with JFH1 RNA and treated with the two drugs were lysed, and the TCID_50_ values of intracellular HCVcc were determined. For both triacsin C and YIC-C8-434, TCID_50_ values were decreased by about 5-fold for intracellular virus (Fig. 3*B*). Hence, although the compounds may partially impair viral RNA replication, they have a more pronounced impact on assembly of infectious virus.

##### Antiviral Mechanisms of Action of Triacsin C and YIC-C8-434

**FIGURE 4.**
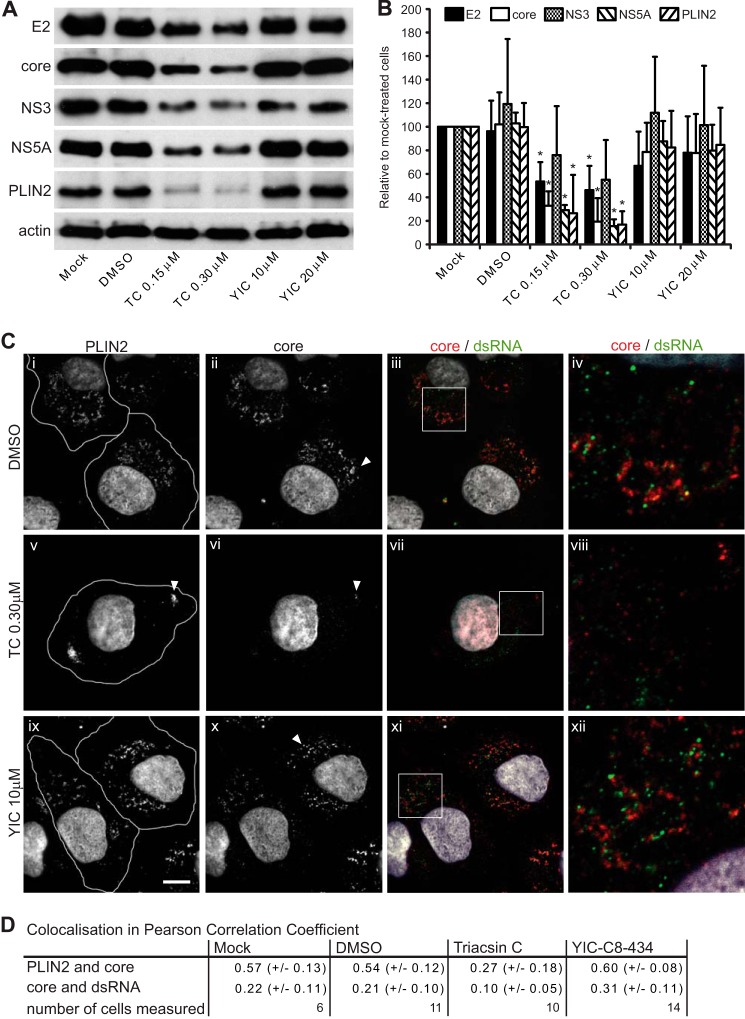
**The effect of triacsin C and YIC-C8-434 on viral proteins.** JFH1 RNA was electroporated into Huh-7 cells for 48 h followed by 29 h of incubation with DMSO, triacsin C (*TC*), or YIC-C8-434 (*YIC*). In *A*, the cells were lysed for SDS-PAGE and Western blot analysis using antibodies against the indicated viral and cell proteins. *B* shows quantification of the data from three independent experiments as determined by densitometry. *Error bars* represent S.D., and * indicates *p* < 0.05 compared with DMSO-treated samples. In *C*, cells were fixed and stained for PLIN2 (*panels i*, *v*, and *ix*), core (*panels ii*, *vi*, and *x*; in *red* in *panels iii*, *vii*, and *xi*), and dsRNA (in *green* in *panels iii*, *vii*, and *xi*). The highlighted *boxes* in *panels iii*, *vii*, and *xi* are shown as higher magnifications in *panels iv*, *viii*, and *xii*, respectively. Cell boundaries are indicated in *panels i*, *v*, and *ix*, and the *scale bar* in *panel ix* represents 10 μm. In *D*, co-localization is shown by Pearson correlation coefficient where −1 means exclusion, 0 is random localization, and +1 represents complete overlap between two signals.

To explore the possible mechanisms responsible for inhibition of intracellular assembly by triacsin C and YIC-C8-434, we first examined the impact of the two drugs on the abundance of a series of viral proteins as well as PLIN2, a cellular factor located at the surface of LDs. Two of these viral proteins, core and NS5A, also are found at the surface of the storage organelles, whereas the other two HCV species examined, E2 and NS3, are targeted to the endoplasmic reticulum membrane. JFH1 RNA was electroporated into Huh-7 cells, which were subsequently treated with triacsin C and YIC-C8-434. After incubation for 29 h, cell extracts were prepared. Western blot analysis showed that PLIN2 levels were reduced by more than 70%, particularly at the higher concentration of triacsin C (Fig. 4, *A* and *B*), whereas YIC-C8-434 had very little effect on the abundance of the protein. These results are consistent with the overall reduction in LDs in triacsin C-treated cells compared with YIC-C8-434 (Fig. 2*B*). We found similar patterns for HCV core and NS5A proteins with levels of core as much as 5-fold lower following triacsin C treatment (Fig. 4, *A* and *B*). By contrast, E2 and NS3 were reduced to a much lesser extent, reflecting the lower HCV RNA synthesis induced by triacsin C, which would affect the abundance of translated proteins from viral genomes. These results are consistent with a link between the presence of intracellular LDs and stability of viral and cellular factors that bind to them. Thus, the impairment of virus assembly by triacsin C is likely to arise from the reduction in numbers of LDs and associated lack of stability of core and NS5A proteins. By contrast, YIC-C8-434 did not mediate any reduction in the viral proteins and therefore presumably decreases virus assembly by a different mechanism (45, 53).

Having established that YIC-C8-434 did not lower the stability of core protein, we examined the effect of the drug on its intracellular distribution by indirect immunofluorescence. In parallel, we also analyzed cells treated with triacsin C. Huh-7 cells were electroporated with JFH1 RNA and then treated with the two drugs as described above. Following drug treatment, cells were fixed and probed with antibodies against core, PLIN2, and dsRNA, which has been used previously to identify putative sites of HCV RNA replication (25). In cells treated only with DMSO, core and PLIN2 co-localized, indicating the presence of core on the surface of LDs (Fig. 4, *C*, *panels i* and *ii*, *white arrows*, and *D*). We also found dsRNA distributed as cytoplasmic punctate foci (Fig. 4*C*, *panel iii*). In the presence of triacsin C, PLIN2 was detected in a low proportion of cells, consistent with the reduction in LDs (Fig. 4*C*). PLIN2 in such cells tended to be distributed toward the cell periphery (Fig. 4*C*, *panel v*, *white arrow*), which would coincide with the remaining LDs found in triacsin C-treated cells shown in Fig. 2. HCV core protein was barely detected (Fig. 4*C*, *panel vi*) in agreement with the reduced levels identified by Western blot analysis (Fig. 4*A*). By contrast, we found that there was no apparent reduction in the number of punctate sites recognized by the dsRNA antibody, indicating the small effect of the drug on HCV RNA replication (Fig. 4*C*, *panel vii*). In cells treated with YIC-C8-434, we did not find any significant reduction in the signals detected for either PLIN2 or core, and the two proteins co-localized to the same extent as with DMSO treatment (Fig. 4*C*, *panels ix* and *x*, *white arrow*). This similarity in co-localization between DMSO- and YIC-C8-434-treated cells was confirmed by determining Pearson correlation coefficient measurements (Fig. 4*D*). We conclude therefore that the distribution of core protein is not altered upon YIC-C8-434 treatment despite the larger size of the LDs. Moreover, the dsRNA antibody detected punctate sites, similar to those in DMSO-treated cells, that co-localize or are in close proximity to core (Fig. 4, *C*, *panels iv* and *xii*, and *D*).

##### Triacsin C but Not YIC-C8-434 Lowers the Specific Infectivity of Virus Released from Infected Cells

**FIGURE 5.**
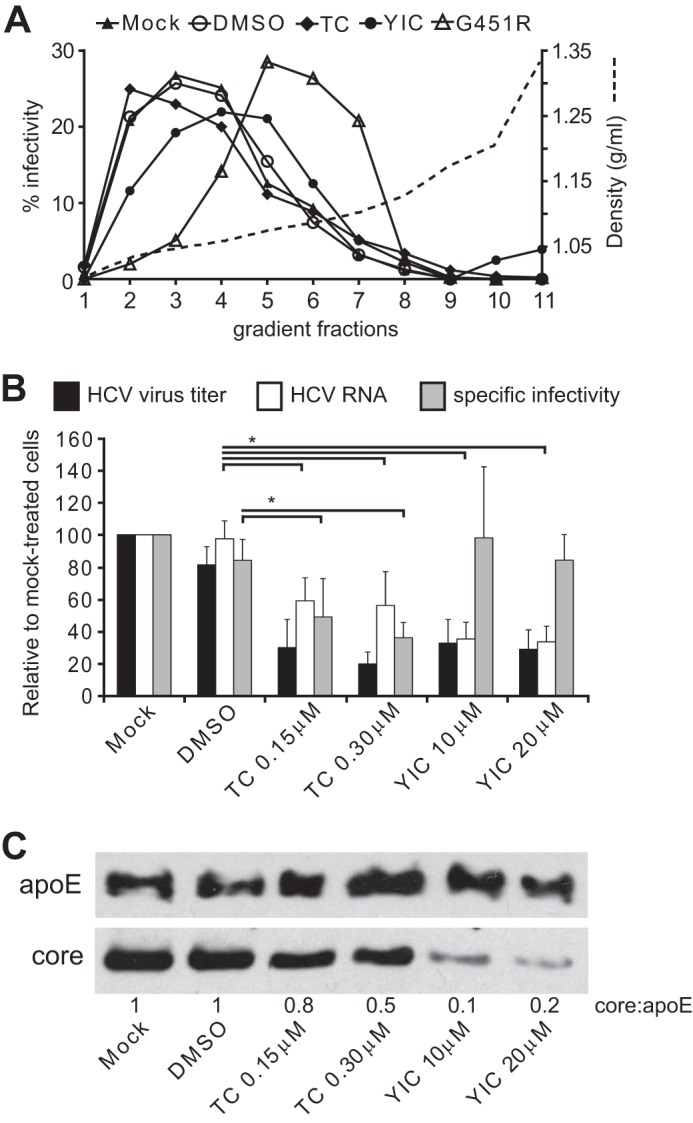
**Biophysical characteristics and specific infectivity of virus particles after treatment with triacsin C and YIC-C8-434.** *A*, 48 h after electroporation with JFH1 or JFH1/G451R RNA, Huh-7 cells were mock-treated (*Mock* and *G451R*) or incubated with DMSO, 0.3 μm triacsin C (*TC*), or 20 μm YIC-C8-434 (*YIC*) for 29 h. Medium from the last 24 h was collected and subjected to ultracentrifugation. Pellets containing virus particles were applied to the top of a continuous 10–40% iodixanol gradient. After centrifugation, 11 fractions (*1–11*) were taken from the top; fraction 1 is the top fraction, and fraction 11 is the bottom fraction. The *dashed line* indicates the density of the gradient. Virus titers were determined for all fractions by measuring secreted embryonic alkaline phosphatase activity in Huh-7/J20 cells. This figure shows the percentage of each fraction relative to total infectivity of all fractions for each individual treatment. *B*, viral RNA and specific infectivity of virus particles released from cells treated with triacsin C and YIC-C8-434. Cells were treated as described in *A*. Extracellular viral RNA (*white bars*) was determined by absolute RT-qPCR, and virus titer was determined by TCID_50_ analysis (*black bars*). Specific infectivity determined from viral RNA and virus titer is shown in *gray bars* (*n* = 3). *Error bars* represent S.D., and * represents *p* < 0.05. *C*, release of apoE and HCV core protein from infected cells was determined by Western blot analysis; virus was concentrated as described under “Experimental Procedures” prior to Western blot analysis. Cells were treated with triacsin C and YIC-C8-434 as described in *A. Numbers* below the blots indicate the ratio of core to apoE as determined by densitometry.

To further examine the possible impact of triacsin C and YIC-C8-434 on infectivity, the physical characteristics of extracellular virus following treatment of infected cells with the two drugs were analyzed by buoyant density centrifugation. HCVcc released from cells treated with the drugs were layered onto 10–40% iodixanol density gradients, and gradient fractions were analyzed for infectivity following centrifugation (Fig. 5*A*). For comparative purposes, we included virus released from mock- and DMSO-treated cells as well as HCVcc produced by a variant that encoded a glycine to arginine mutation at amino acid residue 451 in the E2 glycoprotein (JFH1/G451R). Previous studies have shown that the peak of infectivity for this variant has higher buoyant density as compared with WT HCVcc (54, 55). For WT HCVcc virions produced in either mock- or DMSO-treated cells, peak infectivity had a buoyant density of between 1.03 and 1.06 g/ml. By contrast, the buoyant density of JFH1/G145R virions was higher at between 1.08 and 1.10 g/ml. We found that the peak of infectious HCVcc made in the presence of YIC-C8-434 shifted to a higher buoyant density compared with WT JFH1 (1.05 to 1.08 g/ml), whereas triacsin C did not shift the density of particles from that for WT virus. This alteration in buoyant density is likely to result from a change in the lipid content of virus particles released from cells treated with YIC-C8-434. However, determining the lipid content of virions purified by both buoyant density centrifugation and affinity purification has not proven possible (56).

To further examine virus released from infected cells, the specific infectivity of virions made in the presence of the two drugs was determined by comparing TCID_50_ values with the amount of viral RNA released from cells (Fig. 5*B*). We found that for virus released from triacsin C-treated cells TCID_50_ values were reduced to a greater extent compared with extracellular viral RNA; the amount of extracellular RNA released from triacsin C-treated cells was less than for mock or DMSO treatment, but this reduction correlated with the modest impairment of viral RNA synthesis mediated by triacsin C (see Fig. 3*A*). Hence, the specific infectivity of virus released from triacsin C-treated cells was reduced compared with either mock- or DMSO-treated cells. Previous studies have shown that most of the viral RNA found in the supernatant of infectious cultures from strain JFH1 is encapsidated in non-infectious particles that contain core protein (57, 58). From Western blot analysis of pelleted material from infected cells, the amount of core protein in extracellular material was slightly lowered by triacsin C (Fig. 5*C*), which fits with the reduction in extracellular viral RNA. We concluded that the lower specific infectivity mediated by triacsin C did not arise from impaired release of viral RNA but release of particles with lower infectivity. YIC-C8-434 treatment resulted in even less extracellular viral RNA and a concomitant reduction in core protein (Fig. 5, *B* and *C*). This lowering of viral RNA does not arise from any effect of the drug on viral RNA synthesis and therefore is presumed to result from an inhibition of particle assembly and release upon YIC-C8-434 treatment. Combined with the TCID_50_ values calculated for virus released from YIC-C8-434-treated cells, the specific infectivity of virus was similar to that for mock treatment (Fig. 5*A*). Hence, YIC-C8-434 treatment appears to block virus production, but the released virions have similar infectivity compared with those from mock-treated cells. These effects on virus generated in the presence of triacsin C and YIC-C8-434 appear unrelated to lipoprotein release as the abundance of apoE found in supernatants did not change upon treatment of cells with either drug (Fig. 5*C*).

## DISCUSSION

In this report, we demonstrate that disrupting the pathways that synthesize the major lipid species of LDs leads to a reduction in production of infectious HCV. Our approach utilized two different compounds that had distinct effects on LDs. Hence, the mechanisms for disrupting virion assembly are likely to differ between the compounds, illustrating the intimate but somewhat complex link between HCV and LDs.

The lipid analysis shown in Fig. 1 demonstrates clear differences in the effects of triacsin C and YIC-C8-434 upon cellular lipids. Although both inhibitors brought about a decrease in CEs, triacsin C also reduced mono-, di-, and triglycerides. The reduction in TAGs, DAGs, and CEs by triacsin C is to be expected as the inhibitor reduces the abundance of acyl-CoA, which is necessary for their biosynthesis. However, the change in MAGs is more difficult to explain directly. MAG is generated in cells through TAG and DAG hydrolysis, and thus its decrease is presumably a reflection not just in synthesis of DAGs and TAGs but also in their turnover. Examination of the acyl chain structures of TAGs, DAGs, and MAGs suggests that the addition of oleoyl groups to MAGs and subsequently TAGs is selectively inhibited by triacsin C. Similarly, it would appear that oleoyl incorporation into CEs is inhibited by triacsin C, although this did not reach statistical significance. The lipid species analysis demonstrated that the reduction in CEs by YIC-C8-434 is a largely general effect across all species except for 16:0 and 18:0 CEs, which showed small rises in their relative proportions.

The different effects upon lipid levels by the two inhibitors reveal apparent distinct roles for TAGs and CEs in LD structure. In the triacsin C-treated cells where both TAG and CE levels were reduced, there was a clear loss of LDs. However, the reduction in CEs in the YIC-C8-434-treated cells brought about a reduction in LD number and an increase in their size. The mechanism underlying this affect on the physical characteristics of LDs is not known but could arise from changing the balance among CEs, acylglycerols, and CH. We did observe small increases in MAGs, DAGs, and CH in YIC-C8-434-treated cells. Such changes in the relative ratios of intracellular lipids may induce alterations in the physical properties of LDs, leading to an increase in size. These larger, CE-depleted LDs are clearly less efficient in promoting viral maturation. It is possible that this is due to a distinct alteration in the surface structure of droplets in addition to a change in their size that leads to impairment in virus assembly. Alternatively, droplet CEs may play a key role in virus maturation. The latter hypothesis is supported by the increased density of virus particles from YIC-C8-434-treated cells, suggesting that there is an altered association between virions and cellular lipids from LDs in such cells. Another possibility is that the altered size in LDs induced by YIC-C8-434 may affect the trafficking of LDs, which we previously demonstrated is linked to virion production (59).

Both inhibitors had small but reproducible suppressive effects on HCV RNA replication, although inhibition by triacsin C was more pronounced than that by YIC-C8-434. Previous studies have used acetyl-CoA carboxylase, fatty-acid synthase, and DGAT1 inhibitors to suppress fatty acid biosynthesis and assess their impact on HCV RNA replication (12, 33, 51, 52). Both acetyl-CoA carboxylase and fatty-acid synthase function at the earliest stages of *de novo* fatty acid production, whereas DGAT1 acts at the final stage of TAG synthesis. Inhibition of DGAT1 has no effect on viral RNA replication, whereas blocking either acetyl-CoA carboxylase or fatty-acid synthase activity reduces HCV genome synthesis by at least 50% (12, 33, 51, 52). In the cholesterol pathway, inhibition of the key regulator HMG-CoA reductase with statins also impairs HCV RNA replication (11). Collectively therefore, replication requires lipid species that are synthesized at early stages in TAG and CE production.

Triacsin C inhibits acyl-CoA synthetase, thereby blocking production of fatty acyl-CoA, which is an essential substrate for phospholipid and TAG synthesis as well as esterification of CH in the CE pathway. Labeling studies have demonstrated that, although *de novo* phospholipid synthesis from glycerol is inhibited by triacsin C, there is apparent selective channeling of available fatty acyl-CoAs toward incorporation into phospholipid (39). Hence, phospholipid levels are less affected by triacsin C compared with its impact on the abundance of TAGs. Any slight impairment in phospholipid synthesis by triacsin C could disrupt the composition of membranes, leading to a reduction in viral RNA synthesis at the endoplasmic reticulum. Recent evidence for the importance of acyl-CoA synthetase in the replication of positive strand RNA viruses has been highlighted from studies on poliovirus where infection induces increased acyl-CoA synthetase activity to facilitate the membrane alterations needed for creating sites of RNA replication (60). In contrast to triacsin C, YIC-C8-434 blocks only conversion of CH to CEs through inhibiting acyl-CoA:cholesterol acyltransferase activity (40). It is possible that any subtle variation in the CH:CE ratio or the flux of CH through this pathway may disturb the balance of sterols in the cell and affect membrane fluidity. Such changes could in turn partially modulate replication complex formation or viral protein mobility, leading to the modest decrease in viral RNA levels.

Triacsin C efficiently cleared Huh-7 cells of detectable LDs and thereby reduced virion assembly by about 3-fold. We found that the compound also significantly lowered the levels of viral core and NS5A proteins compared with E2 and NS3. Both core and NS5A are targeted to the surface of LDs, in particular core, which is almost exclusively detected on the storage organelles for strain JFH1 (27). From previous studies, introduction of mutations into core that disrupt localization to LDs also destabilizes the protein (45, 53). Such a loss in the stability of core through mutational insertion could result from improper folding (61) and not necessarily a failure to bind to LDs. In our studies, decreasing the number of LDs in cells by triacsin C treatment was sufficient to target core for proteolysis. Hence, our findings formally demonstrate that core requires LDs as a platform to maintain protein stability. Such a requirement for binding to the surface of LDs as a mechanism to enable stability has also been found for other LD-associated proteins such as PLIN1 and PLIN2, which are degraded upon displacement from the storage organelles (62–65). In our experiments, depletion of LDs also led to a decrease in PLIN2 protein levels. Therefore, our results are consistent with the general notion that the stability of LD-associated proteins is intrinsically linked to the presence of the organelles. Parenthetically, LDs also act as a junction for regulating protein stability through the ubiquitin-proteasome pathway. Among the proteins regulated in this manner is apoB, a major component of VLDL (66, 67). Thus, LDs not only act as storage organelles for lipids but also as compartments that regulate the balance between storage and degradation of proteins (66–69). Presumably, triacsin C-mediated depletion of LDs disrupts these regulatory mechanisms and perhaps exposes LD-associated proteins to alternative degradative pathways. Ultimately, the consequence of removing LDs is a decrease in the abundance of core, which would abrogate virus particle formation.

Compared with triacsin C, YIC-C8-434 did not lead to loss of LDs but increased their overall size. Nonetheless, treatment of cells with the compound reduced virion production. We did not detect any apparent decrease in the abundance of core, and it retained its distribution on the surface of LDs. Therefore, the mechanism for impairing virus assembly is likely to be different from that for triacsin C. Despite the presence of core on LDs, intracellular infectivity was reduced in YIC-C8-434-treated cells, indicating a defect in assembly of infectious virus. Targeting of core to LDs is mediated by a C-terminal domain, termed D2. We have found that decreasing the mobility of the D2 domain at the surface of LDs can block virion production.^4^ We examined D2 mobility in the presence of YIC-C8-434 but did not detect any differences compared with untreated cells (data not shown). Moreover, secretion of apoE, which plays a major role in VLDL assembly, did not decrease upon YIC-C8-434 treatment (Fig. 5*C*), suggesting that lipoprotein secretion is similar in CE-depleted cells compared with normal cells. These data are in agreement with studies on LDL receptor and acyl-CoA:cholesterol acyltransferase 2 knock-out mice, which have a similar amount of VLDL secretion as compared with LDL receptor-only knock-out mice. Interestingly, the double knock-out mice secreted VLDL with depleted levels of CEs but slightly increased TAGs (70). CE depletion by YIC-C8-434 may alter the lipid composition of virus particles that are assembled as part of the cellular VLDL pathway. Indeed, lipidomic analysis of affinity-purified virus particles suggests that CEs and cholesterol are major lipids in virions (56). Formal demonstration that the lipid content of infectious virus particles is altered by either YIC-C8-434 or triacsin C would require isolation of purified virions from density gradients; however, such an approach has proven technically challenging because of low yields that are not feasible for biochemical studies (56).

Although we were unable to examine the composition and morphology of virions produced in the presence of the two drugs, we did examine the specific infectivity of the virus particles released from cells. Virions produced in the presence of triacsin C had lower specific infectivity, whereas infectivity was not affected by YIC-C8-434 compared with mock-treated cells. This suggests that depleting cells of LDs increases the ratio of non-infectious particles released from cells compared with those that are infectious. Hence, there may be more than one route for release of particles containing viral RNA and protein, but the presence of LDs enhances production of infectious virions. By contrast, YIC-C8-434 does not affect specific infectivity but does decrease the overall numbers of viral particles released. This could be regulated by increased size of LDs and their disengagement from cell pathways that are necessary for the entire assembly and release process. We have shown previously that restricting the mobility of LDs reduces virus assembly (59). It is possible that those LDs, which would typically participate in virion assembly and release, may be sequestered in YIC-C8-434-treated cells. Such a situation would not affect either core localization or stability but could reduce virion assembly.

Our studies formally demonstrate the importance of LDs to the assembly pathway for HCV virions. The two compounds used in the study had quite distinct effects on LDs, but it is perhaps worth noting that the quantitative changes in total CEs and TAGs were not dramatic. Therefore, even subtle changes in the cell lipidome can have significant effects on virus assembly. The use of acyl-CoA:cholesterol acyltransferase inhibitors in particular to treat atherosclerosis or hypocholesterolemia has been explored in the past with little success (71, 72). There is renewed interest in such compounds because of their potential as anticancer agents (73). Thus, any agents that target synthesis of TAGs and CEs with therapeutic value, especially in the development of HCV-associated hepatocellular carcinoma, could display antiviral properties as well as anticancer properties. This would expand the options for treating chronic HCV infection, especially in patients with serious liver disease who frequently respond less well to current therapies.

## Supplementary Material

Supplemental Data

## Footnotes

4 A. Filipe, R. Montserret, S. Boulant, F. Penin, and J. McLauchlan, manuscript in preparation.

## Footnotes

3 The abbreviations used are:

HCV: hepatitis C virus
CH: cholesterol
CE: cholesterol ester
DAG: diacylglyceride
LD: lipid droplet
MAG: monoacylglyceride
TAG: triacylglyceride
DGAT1: diacylglycerol acyltransferase 1
dsRNA: double-stranded RNA
PLIN: Perilipin
TCID_50_: tissue culture 50% infectious dose
qPCR: quantitative PCR
ESI: electrospray ionization
HCVcc: *cell culture-derived HCV*.
