# Supplementary material for: Modulation of Triglyceride and Cholesterol Ester Synthesis Impairs Assembly of Infectious Hepatitis C Virus
Source: J Biol Chem. 2014 Jun 10;289(31):21276–88. doi: 10.1074/jbc.M114.582999 (PMC4118089; doi:10.1074/jbc.M114.582999)
Supplement: Supplemental Data [file supp_289_31_21276__index.html]

Modulation of Triglyceride and Cholesterol Ester Synthesis Impairs Assembly of Infectious Hepatitis C Virus — HCV Virion Assembly and Lipid Synthesis — Supplemental Data 

# Modulation of Triglyceride and Cholesterol Ester Synthesis Impairs Assembly of Infectious Hepatitis C Virus

## Supplemental Data

**Files in this Data Supplement:**

- Supplementary Figures
