# Supplementary material for: Modulation of Triglyceride and Cholesterol Ester Synthesis Impairs Assembly of Infectious Hepatitis C Virus
Source: J Biol Chem. 2014 Jun 10;289(31):21276–88. doi: 10.1074/jbc.M114.582999 (PMC4118089; doi:10.1074/jbc.M114.582999)

SUPPLEMENTARY FIGURES

SUPPLEMENTARY FIGURE 1. Viability of cells at different concentrations of Triacsin C and YIC-C8-434 over a time course. The viability of cells was determined using a resazurin assay, a fluorometric method for estimating the number of viable cells. Briefly, cells in 24-well plates were either mock-treated or incubated with Triacsin C and YIC-C8-434 for the indicated times. Culture medium was removed and the cells were incubated with resazurin (20 µg/ml) for between 1-4 h. 200 µl of the resazurin was transferred into a 96-well opaque-walled plate and fluorescence between 560-590 nm was measured.

SUPPLEMENTARY FIGURE 2. Morphologic changes to LDs after treatment with Triacsin C and YIC-C8-434. Huh-7 cells were mock-treated (panel v) or incubated with 0.15 µM or 0.3 µM Triacsin C (TC) (panels vi and vii, respectively) or 10 µM YIC-C8-434 (panel viii) for 24 h, followed by fixation and staining for LDs and nuclei using LD540 (red) and DAPI (blue) respectively. LDs in the periphery of the cell are indicated with an arrowhead. Panels i-iv show the images recorded by DIC for the corresponding stained images in panels v-viii. The scale bars in panel i and v represent 10µm.

Supplementary Figure 1

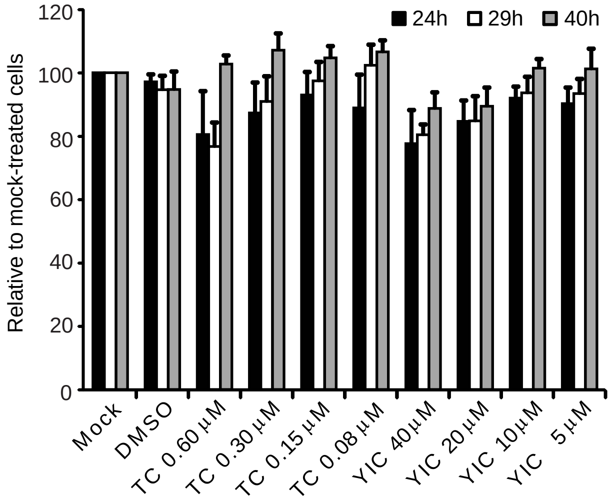

Supplementary Figure 2

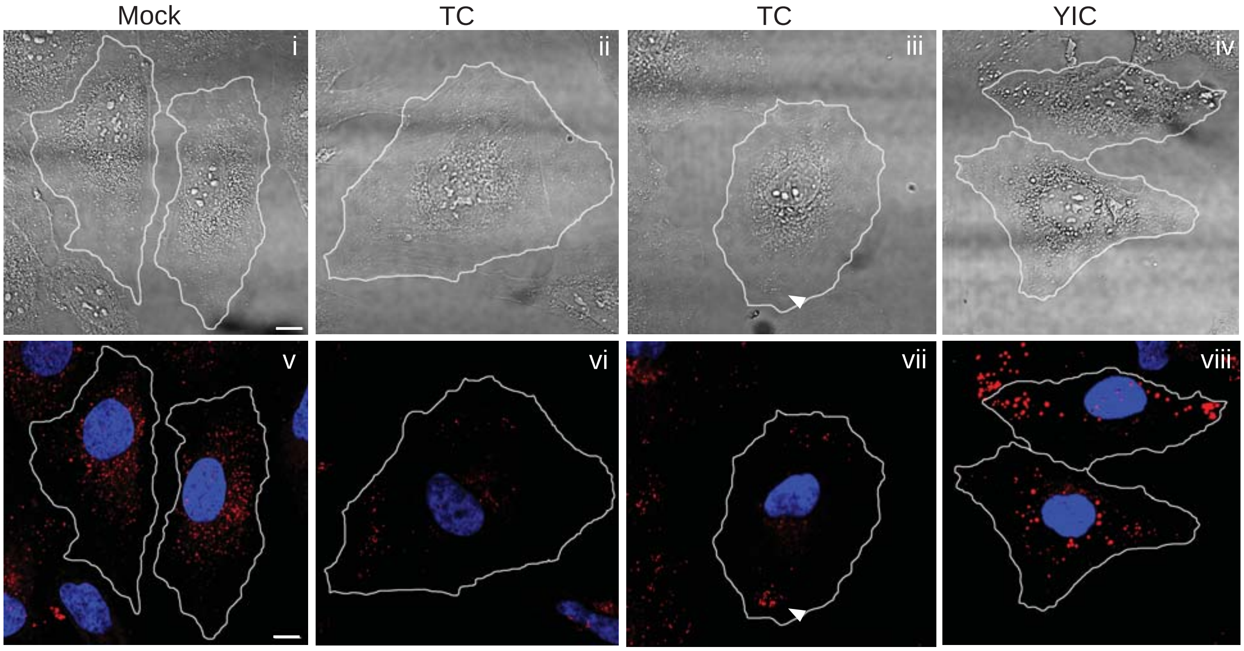

Supplement: Supplemental Data [file supp_M114.582999_jbc.M114.582999-1.pdf]
